# Health Benefits of Polyphenols and Carotenoids in Age-Related Eye Diseases

**DOI:** 10.1155/2019/9783429

**Published:** 2019-02-12

**Authors:** Simona Bungau, Mohamed M. Abdel-Daim, Delia Mirela Tit, Esraa Ghanem, Shimpei Sato, Maiko Maruyama-Inoue, Shin Yamane, Kazuaki Kadonosono

**Affiliations:** ^1^Pharmacy Department, Faculty of Medicine and Pharmacy, University of Oradea, Oradea, Romania; ^2^Pharmacology Department, Faculty of Veterinary Medicine, Suez Canal University, Ismailia, Egypt; ^3^Department of Ophthalmology and Micro-technology, Yokohama City University, Yokohama, Japan; ^4^Faculty of Medicine, Al-Azhar University, Cairo, Egypt

## Abstract

Oxidative stress and inflammation play a critical role in the initiation and progression of age-related ocular abnormalities as cataract, glaucoma, diabetic retinopathy, and macular degeneration. Therefore, phytochemicals with proven antioxidant and anti-inflammatory activities, such as carotenoids and polyphenols, could be of benefit in these diseases. We searched PubMed and Web of Science databases for original studies investigating the benefits of different carotenoids and polyphenols in age-related ophthalmic diseases. Our results showed that several polyphenols (such as anthocyanins, *Ginkgo biloba*, quercetin, and resveratrol) and carotenoids (such as lutein, zeaxanthin, and mezoxanthin) have shown significant preventive and therapeutic benefits against the aforementioned conditions. The involved mechanisms in these findings include mitigating the production of reactive oxygen species, inhibiting the tumor necrosis factor-*α* and vascular endothelial growth factor pathways, suppressing p53-dependent apoptosis, and suppressing the production of inflammatory markers, such as interleukin- (IL-) 8, IL-6, IL-1a, and endothelial leucocyte adhesion molecule-1. Consumption of products containing these phytochemicals may be protective against these diseases; however, adequate human data are lacking. This review discusses the role and mechanisms of polyphenols and carotenoids and their possible synergistic effects on the prevention and treatment of age-related eye diseases that are induced or augmented by oxidative stress and inflammation.

## 1. Introduction

Age-related ophthalmic diseases as cataract, age-related macular degeneration (AMD), glaucoma, and diabetic retinopathy are the main causes of progressive and irreversible vision loss worldwide [1–3]. These pathologies are joined by dry eye disease (DED), a prevalent eye disorder that affects the elderly population [4]. The pathogenic processes of these diseases are complex and unclear and sometimes depend on numerous factors. Unfortunately, most of these conditions are diagnosed in advanced stages at which effective treatments are not available [5]. As a result, both the improvement of diagnostic and therapeutic approaches and the prevention of age-related eye diseases have become global health priorities.

For this purpose, researchers have investigated the health benefits of several strategies to maintain visual acuity and prevent degenerative eye conditions [6, 7]. Experimental studies have found that fruit and vegetable consumption contributes to preserving vision and even reversing visual impairment [8, 9]. These beneficial effects have been attributed to the presence of some phytochemical compounds with bioactive properties, such as polyphenols and carotenoids [6, 10].

The pathophysiology of several eye diseases involves oxidative stress, and therefore, the antioxidant activity of phytochemicals is of particular importance. Carotenoids and polyphenols are plant-based molecules that have shown potent antioxidant and anti-inflammatory activities in several animal models of disease [11–15]. This review deals in depth with the role of antioxidant polyphenol and carotenoid properties in the prevention and treatment of age-related ophthalmic diseases, whose pathogeneses involve oxidative stress and inflammation.

### 1.1. Oxidative Stress: Implications for Age-Related Eye Diseases

Oxidative stress occurs due to imbalance in the redox hemostasis between prooxidant and antioxidant systems [16] and is involved in the pathogenesis of several inflammatory, degenerative, neoplastic, and cardiovascular disorders [17, 18]. Antioxidant enzymes (such as SOD, CAT, and GPx) can neutralize the production of toxic oxygen species, thus normalizing the body homeostasis [19]. Oxidative stress results from the inability of antioxidant enzymes to remove free radicals [20]. This imbalance leads to oxidative alteration of cellular targets, such as proteins, lipids, and DNA structures [18], ending in apoptosis or cellular necrosis [21]. A frequent risk element in numerous degenerative diseases in which oxidative stress plays a major role is aging. As humans advance in age, the rate of oxidative stress-induced cellular malfunction and death exceeds the rate of cellular regeneration [22, 23].

**Figure 1:**
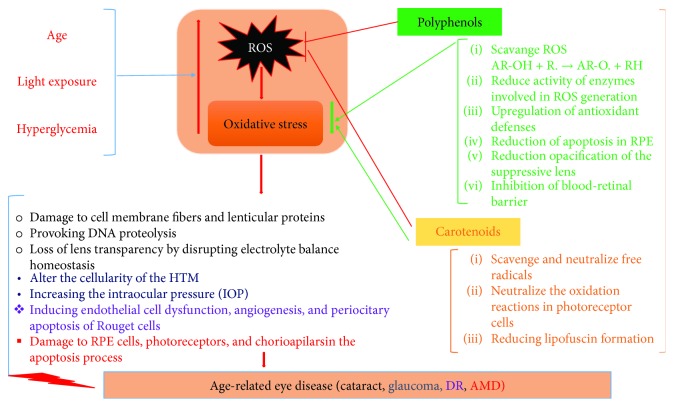
Summarization of the implications of oxidative stress in age-related ocular diseases and effects of phytochemicals.

The eye is particularly vulnerable to oxidative stress due to its exposure to light, rich content of mitochondria, and high metabolic rate of photoreceptors. Due to the disequilibrium between the production and neutralization of reactive oxygen species (ROS), it results in oxidation of cellular constituents and ultimately malfunctions and degeneration of retinal tissues [24].  Figure 1 summarizes the implications of oxidative stress in age-related ocular diseases.

### 1.2. Cataract

In developing countries, cataract is the most prevalent cause of blindness and visual impairment in the elderly people. Currently, the management of a visually significant cataract is primarily surgical, which is the only available option to ophthalmologists. The pathogenesis of cataract involves Na^+^/K^+^ adenosine triphosphatase activity changes, oxidative stress, lens protein aggregation, polyol pathway activation, advanced glycation end-products, and genetic anomalies [25]. The contribution of ROS towards cataract pathogenesis occurs through (1) damage to cell membrane fibers and lenticular proteins, (2) provoking DNA proteolysis, and (3) loss of lens transparency by disrupting electrolyte balance homeostasis [26, 27].

### 1.3. Glaucoma

Glaucoma is an optical neuropathy, characterized by progressive degeneration of the retinal ganglion cells. The degeneration of these cells, which are actually neurons of the central nervous system (CNS) with axons in the optic nerve, causes progressive optical atrophy and irreversible visual loss [28]. In addition to ganglion cell loss, most glaucoma types are characterized by high intraocular pressure [29]. The diagnosis of open-angle glaucoma is often delayed due to the fact that its progression may be asymptomatic until a relatively late stage [30]. Several factors contribute to glaucoma pathophysiology, including aging, genetic predisposition, and exogenous environmental and endogenous factors.

Reactive oxygen species may damage the cells within the human trabecular meshwork (HTM), especially its endothelial cells. This consequently slows the drainage of the aqueous humor, increasing the intraocular pressure (IOP) [31]. Several studies reported oxidative stress and low levels of antioxidants as early events in patients with glaucoma [12, 32]. This is further exacerbated by the upregulated expression of the endothelial leukocyte adhesion molecule due to oxidative stress and activation of the interleukin-1 inflammatory cytokine [33–35]. Glaucoma cannot be cured; however, its progression may be delayed or prevented by reducing the IOP [36, 37]. The interest in investigating phytochemicals due to their antioxidant and anti-inflammatory properties has opened new treatment options with reduced side effects [34, 38].

### 1.4. Diabetic Retinopathy

Diabetic retinopathy (DR) is a major complication of diabetes mellitus. In the industrialized world, it is regarded as the leading cause of blindness in individuals who have not reached the retirement age [39]. Many interconnected mechanisms are implicated in the complex pathogenesis of DR, including oxidative stress [39]. Hyperglycemia induces many metabolic abnormalities in the retina, producing ROS, which are involved in the subsequent induction of oxidative stress [40]. Usually, this oxidative stress causes retinal lesions (inducing endothelial cell dysfunction, angiogenesis, and peri-ocitary apoptosis of Rouget cells/or mural cells) [40].

Oxidative stress plays a central role in DR development and in its critical phases: proliferative diabetic retinopathy (PDR) and diabetic macular edema (DME) [39]. The most glaring characteristics of DR are the vascular disturbances and retinal blood barrier (RBB) disturbance; both can be caused by oxidative stress [27, 41]. Consequently, their inhibitors may have protective effect on the retina. Antioxidants may be considered beneficial to DR because they reduce ROS production, neutralize free peroxide, and enhance the antioxidant defense system [40].

### 1.5. Age-Related Macular Degeneration (AMD)

This is a progressive, age-related degeneration of the underlying retinal pigment epithelium (RPE) and the retinal macula [42]. Although it is presumed that the cumulative repercussions of oxidative stress over years may be the first stimulus for AMD, the pathogenesis of AMD is yet uncertain [43]. However, RPE cells are considered to be the critical lesion site in AMD [44]. Excessive production and accumulation of ROS play a major role in the pathogenesis of AMD because their levels increase in the aging retina, even if retinal cells and RPE contain numerous antioxidants (enzymatic and nonenzymatic). The high level of ROS and attenuated antioxidant cell defense systems results in oxidative stress, leading to damage and apoptosis of RPE cells, photoreceptors, and chorioapilars [45, 46].

### 1.6. Dry Eye Disease

It is considered to be a multifactorial disease of the ocular surface and the tears that it manifests through visual disturbance, symptoms of discomfort, and tear film instability [47].

Recent laboratory and clinical studies have confirmed that the dry eye may be considered a chronic inflammatory disease and may be initiated by numerous intrinsic and extrinsic factors favoring an unstable and hyperosmolar lacrimal film [48].

Many times, some environmental factors are involved in dry eye disease; of these, we mention the following: exposure to chemical and physical pollutants, ultraviolet radiation (UV) and ozone, and chronic use of preserved eye drops (such as glaucoma treatment). These factors mentioned above increase the potential for oxidative stress and intensify the inflammation of the ocular surface. Phytochemicals with antioxidant and/or anti-inflammatory properties may be considered to be an alternative in the prevention and treatment of this disease [49]. Therefore, in the treatment of ocular disorders linked to oxidative stress, ameliorating the negative effects of ROS has been suggested to be a rational therapeutic strategy.

## 2. Materials and Methods

### 2.1. Identification of Reviewed Studies

We searched Medline (via PubMed) and Web of Science (WoS) databases for original studies that investigated the benefits of phytochemicals in age-related ocular diseases. We used the following keywords with different combinations: Phytochemicals OR Phytonutrient OR Plant-based OR Plant-derived OR Carotenoids OR Xanthophylls OR Lutein OR Polyphenols OR Anthocyanins AND Age-related OR Senile OR Elderly AND Eye disease OR Ophthalmic disease OR Cataract OR Glaucoma OR Macular degeneration OR Diabetic retinopathy OR Dry eye disease.

### 2.2. Study Selection

No restrictions by publication language or timing were applied. The retrieved studies were screened for eligibility. Overall, 2330 unique records were retrieved and screened for eligibility. Relevant findings were extracted and organized in a narrative approach.

## 3. Results and Discussion

### 3.1. Role of Polyphenols in the Prevention and Treatment of Age-Related Ophthalmic Diseases

**Figure 2:**
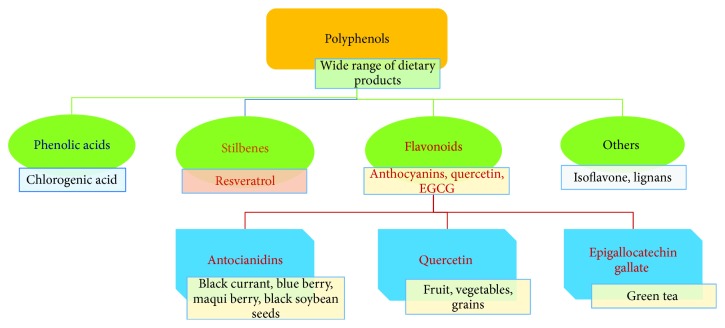
Classification of main polyphenols investigated for age-related eye diseases and their natural sources.

Polyphenols represent a broad group of phytochemicals with over 10000 different compounds [50], many of which have proven health benefits: antioxidant, anti-inflammatory, antiallergic, antimicrobial, and antiviral effects [51, 52]. They are classified into several groups based on their carbon backbone [53] as it is illustrated in Figure 2.

The beneficial effects of polyphenols include: scavenging free radicals, ameliorating inflammation, and improving ocular blood flow and signal transduction [54]. There are recent studies describing new findings regarding the beneficial effect of polyphenol consumption on ocular health (reduction of apoptosis in the RPE, opacification of the suppressive lens, and inhibition of the blood-retinal barrier) [55].

The retina is highly susceptible to oxidative stress due to its rich content of polyunsaturated fatty acids and oxygen and its heavy exposure to light [56, 57]. In addition, oxidative stress can be involved in the production of severe inflammation by increasing the proinflammatory cytokines in the retinal tissue. These cytokines degrade the RBB and produce vascular cell death and apoptosis through tumor necrosis factor-*α*, chemotactic proteins, intercellular adhesion molecule 1, and interleukin- (IL-) 1*β* [54, 58].

Polyphenols can activate the process of transcription factor Nrf2, Nrf2 being considered to have the main role in protecting the cell from inflammation and oxidative stress [59]. The senso-redox transcription factor Nrf2 has an essential role in regulating the induction of detoxification and/or antioxidant phase II enzymes (HO-1, SOD, etc.); these enzymes have the effect of cellular defense against oxidative stress and exercise of the cytoprotective mechanism [60]. Numerous researches have studied and elucidated the molecular mechanisms considered to be responsible for activating Nrf2. A Kelch-associated Kelch homolog (ECH) associated with Kelch (Keap1)—cytoskeleton-binding protein—binds to Nrf2; it regulates the translocation of the protein to the nucleus, or its activation [61].

Nrf2 binds, after nuclear translocation, to the cis element (consensus called antireceptive element (ARE) or electrophilic response element (EpRE) present in the promoter region of genes encoding numerous antioxidant enzymes); it also binds to other transaction factors as follows: the small Maf-F/G/K, ARE coactivators which include the cAMP response-binding protein (CREB or CBP binding protein), p300, which may coordinate the transcription of the antioxidant gene driven by ARE [62, 63].

The mechanisms implied in the antioxidant activity of polyphenols include suppression of ROS formation (scavenging of ROS, inhibition of enzymes involved in their production, or protection or upregulation of antioxidant defenses) [64, 65]. Polyphenols can reduce the catalytic activity of enzymes involved in ROS generation, therefore reducing oxidative damage [66]. The explanation of the molecular mechanism of induction of the antioxidant enzyme by polyphone is still largely unrealized. There is, however, a universally accepted model for inducing ARE-mediated antioxidative gene expression. This mechanism involves the phosphorylation of serine and/or threonine residues of Nrf2 by protein kinases, resulting in ARE binding and subsequent increased nuclear accumulation of Nrf2 [67].

Some studies asserted that ROS formation increases free metal ions by reducing hydrogen peroxide and generating very reactive hydroxyl radicals. The reduced redox potentials of polyphenols can thermodynamically diminish free radical production by bonding them in chelates with metal ions such as iron and copper [68]. In addition, some polyphenols can inhibit the characteristic uncontrolled ocular angiogenesis in AMD by restoring the retinal structure and increasing the RPE function and choroidal blood flow [69]. By inhibiting oxidative stress and blocking the production of proinflammatory cytokines, polyphenols can attenuate vascular leakage and neovascularization in the retina of diabetic patients [70–72]. The effects of some polyphenols on age-related eye diseases are presented in Tables 1 and 2 and are discussed below.

#### 3.1.1. Anthocyanins

Anthocyanins are flavonoid compounds with multiple biomedical functions [72, 73], including vision improvement [74]. Some recent scientific research clearly suggests that anthocyanins use several different mechanisms of action for biological beneficial effects on health. The free radical scavenging activity and antioxidant capacity are still the most common [75]. It has been shown that anthocyanidin isolates and high bioflavonoid mixtures in anthocyanidin provide several major actions: inhibition of enzymes, protection against DNA cleavage, anti-inflammatory activity, estrogen activity (modulation of the development of symptoms of hormone-dependent disease), stimulation of cytokine production thereby regulating immune responses, peroxidation, decrease of capillary permeability and fragility, and membrane hardening [67].

Several studies were performed to investigate their effects on asthenopia (eye fatigue). While some studies reported significant improvements in visual acuity [75], others [74, 76–78] reported contradictory effects for anthocyanins in this regard. Moreover, several experimental studies have reported antioxidant and protective effects at the eye level for anthocyanins, extracted from various fruits [79–81]. For example, anthocyanins extracted from grape skin were associated with inhibited opacity of the lens in sodium selenite-induced cataract in rats [78]. Anthocyanins from blackcurrant and blueberry extracts have demonstrated antioxidant effects at the level of RPE cells [80] by inhibiting the photooxidation of pyridinium bisretinoid (A2E) molecule and neutralizing oxygen free radicals [80, 81].

In an experimental study by Jang et al. [82], the accumulation of anthocyanins in the eye tissues of animals (after 4 weeks of dietary supplementation with blueberries) exhibited ocular protective effects and reversal of oxidative effects. Other studies performed on rats have shown that anthocyanins from blueberry extract can inhibit diabetes-induced retinal abnormalities, thus preventing DR induction [83, 84]. Moreover, anthocyanins extracted from black soybean seeds have shown their protective effects on the retinal neurons (from structural and functional damage produced by N-methyl-N-nitrosourea in rats) [85]. In vitro studies on Maqui berry extract have shown that it can protect the retinal cells against light-induced photoreceptor degeneration due to its content of anthocyanins [86]. Further *in vivo* and *in vitro* studies will be needed to establish the pleiotropic mechanisms of anthocyanins and to show how they can practically interfere in different visual processes [87, 88].

#### 3.1.2. Epigallocatechin Gallate

Epigallocatechin gallate (EGCG) is the main flavonoid present in green tea, representing more than 50% of the whole amount of polyphenols in this type of tea [89]. Numerous studies indicate that the remarkable antioxidant properties of polyphenols (tea) may be due to the inhibition of ROS-generating enzymes. These enzymes contribute to the production of NO-mediated free radicals. In the study of Wu et al., in addition, the induction of the HO-1 enzyme in endothelial cells was also found due to the activation process of Akt and Nrf2 by EGCG, which gives the protective measure of endothelial cell protection against H_2_O_2_-mediated oxidative stress [90]. Other rodent studies have demonstrated the pleiotropic effect of EGCG. This effect wields a neuroprotective measure by modulation of antioxidative enzyme activity (SOD, GST), associated with suppression of ROS generation and with the protection of neuronal cells (from glycation-induced neurotoxicity) [67].

Some plausible mechanisms of explication of EGCG-induced Nrf2 activation are the oxidation or the modification of cysteine thiols present in Keap1 by ROS and/or via the active form of EGCG (during its redox cycling). However, it may be plausible that EGCH is mediated by ARE-mediated regulation of the antioxidant gene (by activation of MAPK, which ultimately activates Nrf2). However, there are numerous studies that have shown MAPK inhibition by phytochemicals, which eventually caused Nrf2 activity [60, 61].

Green tea leaf extract has been associated with an anticataract effect [91, 92], and short-term supplementation with EGCG has shown beneficial effects on the treatment of glaucoma through the protection of retinal neurons subjected to injuries due to high IOP [93, 94]. Further, it has been shown to suppress vascular endothelial growth factor (VEGF) and extracellular signal-regulated kinase (ERK) 1/2 signaling pathways [95, 96]. Supplements containing green tea, administered to diabetic rats, increase GSH levels and catalyze SOD activities, while reducing VEGF and TNF-*α* values and protecting retinal vasculature and retinal endothelial cells from/against apoptosis [97]. These data also describe possible mechanisms of beneficial effects of green tea on DR treatment [97]. Topical treatment with artificial tears (containing epigallocatechin gallate (EGCG) and hyaluronic acid (HA)) of DED rabbits had anti-inflammatory and mucoadhesive properties [98].

#### 3.1.3. Ginkgo biloba

*Ginkgo biloba* (GB) is a plant that possesses several biological actions that encouraged researchers to investigate its benefits in the prevention or treatment of ocular diseases, especially glaucoma [99]. The GB extract significantly inhibited the steroid-induced increase of intraocular pressure in rabbits and prevented the adverse effects of dexamethasone on the trabecular meshwork cells [100]. Moreover, the GB extract was associated with positive effects on the improvement of deteriorated visual field in patients with normotensive (open-angle) glaucoma [101–107]. Further, its ability to prevent cell membrane damage by free radicals that may aid stop the evolution of AMD was demonstrated [108]. Through the ginkgolide B terpenoid contained in the GB extract, it protects retinal ganglion cells and promotes their growth in vitro by antiapoptosis [109].

#### 3.1.4. Quercetin

Quercetin is a flavonoid present in many fruits and vegetables. Studies have reported that quercetin effectively protects against hydrogen peroxide-induced cataracts and diabetes-induced retinal lesions [110, 111]. It is well known that oxidative stress is the major cause of neurodegenerative accountability, by its action in steering neurotrophic factors and activating apoptosis (in retinal diabetes). Some studies have shown that treatment with quercetin in diabetic rats causes a substantial increase in neurotrophic factor levels and an inhibition of cytochrome activity and a caspase-3 activity in retinal diabetes. Furthermore, the level of an antiapoptotic Bcl-2 protein increases in the diabetic retina treated with quercetin. It can be argued that quercetin can protect neuronal damage in retinal diabetes, ameliorates neurotrophic factor levels, and inhibits neuronal apoptosis [112].

In *in vitro* RPE cells, quercetin inhibits oxidative stress that causes apoptosis as highlighted in experimental studies [113, 114]. Wang et al. demonstrated that quercetin protects the retina subjected to oxidative stress and inflammation, induced by light [115], which could provide new strategies to impede AMD occurrence and progression. The use in artificial tears of quercetin in the experimental model, alone or in combination with resveratrol, suggests that their topical application could be used for DED treatment [116].

#### 3.1.5. Resveratrol

Resveratrol (trans-3,40,5-trihydroxystilbene) is a polyphenolic phytoalexin that belongs to the stilbene family (a group of compounds that consist of 2 aromatic rings joined by a methylene bridge) that is mainly found in grape seed and skin, as well as fruit berries [69]. In a study by Doganay et al. in experimental rats, resveratrol effectively suppressed cataract development after sodium selenite exposure and increased the lenticular levels of reduced glutathione [117]. Moreover, it ameliorated the damages caused by hydrogen peroxide in the lens epithelial cell morphology by inhibiting p53-dependent apoptosis (by activating sirtuin-1) [118].

In another study by Luna et al., resveratrol treatment was associated with a significant downregulation of glaucoma marker expression, caused by chronic oxidative stress in the trabecular meshwork cells through its antiapoptotic effect [69, 119]. Moreover, it suppressed the production of proinflammatory markers, comprising IL-8, IL-6, IL-1a, and endothelial leucocyte adhesion molecule-1 (ELAM-1) [119].

At the retinal level, diabetes-induced oxidative stress, apoptosis, proinflammatory cytokine production, and NF-*κ*B activation have been shown to be inhibited by resveratrol. Destruction of diabetic-induced neuronal cell and vascular hyper permeability were stopped by resveratrol treatment [120–124]. The ocular structure and activity in three patients with AMD were enhanced over a two- to three-year survey, implying the efficiency of resveratrol in AMD treatment [125].

### 3.2. Role of Carotenoids in the Prevention and Treatment of Age-Related Ophthalmic Diseases

Carotenoids are a group of lipo-soluble pigments comprising over 600 natural compounds, divided into three classes of xanthophylls, carotenes, and lycopene [126, 127]. Although they are widespread in nature (plants, fruits, vegetables, fungi, bacteria, algae, and fish) [128, 129], only about 40 carotenoids are present in the human diet, of which 20 were found in tissues and biological fluids [126]. At the level of the human macula, lutein, zeaxanthin, mezoxanthin, and xanthophyll carotenoids are responsible for the yellow pigmentation of the fovea and have been chemically identified [130] A diet high in these carotenoids is associated with a low incidence of age-related eye diseases, such as AMD and cataracts [131, 132].

Lutein and zeaxanthin are obtained exclusively from food and are found especially in vegetables with green leaves and in yellow and orange fruits and vegetables. Meso-zeaxanthin is rarely found in diet and is therefore considered a metabolite of the other ingested carotenoids [133–135]. Due to the conformational similarity between lutein and meso-zeaxanthin [136], it was considered that lutein is the direct precursor of meso-zeaxanthin; a hypothesis which was confirmed by some experimental studies [137, 138]. The presence of meso-zeaxanthin in ocular tissues only (macula, retina, and RPE/choroid) [139] indicates that lutein conversion in meso-zeaxanthin is produced at the eye level.

Carotenoids are very powerful antioxidants that can scavenge and neutralize free radicals, such as hydroxyl radical and superoxide anion [140]. The association of carotenoid supplementation with a lower incidence of ocular diseases is primarily due to the ability of macular carotenoids like zeaxanthin and lutein to neutralize the oxidation reactions in photoreceptor cells [136, 141, 142]. All these eye-protective nutrients undergo the oxidation process and a series of transformations which protect the macula. 3-Hydroxy-*β*,*ε*-caroten-3′-one is a substance that was identified as the direct oxidation product of lutein present in the human eye and in monkey retinas [136].

Another protective function that was reported for carotenoids is reducing lipofuscin formation. With age, the RPE cumulates lipofuscin, which is a fluorescent protein-lipid mixture containing by-products of vitamin A metabolism, along with lipid peroxidation products [143, 144]. This compound is noxious to the mitochondria and was found to induce apoptosis in cultured RPE cells upon exposure to blue light [145–147]. Interestingly, the amount of lipofuscin formed *in vivo* and in cultured RPE cells was found to be diminished in the presence of lutein and zeaxanthin [143, 148]. The effects of macular carotenoids on age-related eye diseases are presented in Table 3.

#### 3.2.1. Lutein, Zeaxanthin, and Meso-zeaxanthin

Through their antioxidant and anti-inflammatory properties and ability to filter blue light, macular pigments (MP) play a major role in reducing the incidence of eye disorders, especially AMD [149, 150]. Several studies have examined the role of MP on visual function, and most have reported a positive influence [151]. It has also been found that the supplementation with all three MP is superior to formulations containing only two nutrients [151].

Epidemiological research and a number of large-scale clinical trials (such as Age-Related Eye Disease Study 2 (AREDS2)) have directed the researchers' attention to the functional benefits and potential ocular health of these three xanthophyll carotenoids, which are consumed through the diet or using supplements [136]. AREDS2 is a randomized, placebo-controlled study designed to demonstrate whether supplementation with lutein (10 mg/day) and zeaxanthin (2 mg/day) can slow the rate of progression of AMD. Addition in primary analyses of lutein + zeaxanthin to the AREDS formulation did not further reduce risk of progression to advanced AMD [152]. A five USA ophthalmology center eye disease case control study demonstrated that a higher dietary intake of some carotenoids (lutein and zeaxanthin) is associated with reduced AMD risk [153].

At the lens level, there are only lutein and zeaxanthin, which protect the eye against photooxidative damage and filter the blue light (which by generating ROS can produce photodamage) [154]. Data on their role in cataract is not unitary; some epidemiological studies have reported an inverse association between cataract risk and macular pigment density [155–158], while others reported no association [159] or positive association [160]. These differences can be explained by the fact that the etiology and evolution of the disease can be dependent on the type of cataract (nuclear sclerotic, cortical, and posterior subcapsular) [161].

Several observational and interventional studies implied that subjects with increased dietary ingestion and/or high plasma concentrations of lutein and zeaxanthin have a lower risk of AMD [162–167]. Moreover, MP supplementation has been shown to enhance macular pigment optic consistency in the reticular pseudodrusen [168, 169], which correlates with a low risk for AMD.

However, other studies failed to record such association [170, 171]. Moreover, various studies [172, 173] showed the function of carotenoids in DR improvement. Zeaxanthin has been demonstrated to suppress diabetes-induced retinal oxidative lesions and increased retinal levels of VEGF and ICAM-1 [173]. Similarly, lutein could diminish oxidative stress and lipid peroxidation in diabetic retinal tissues [174, 175]. Other carotenoids (e.g., lycopene and astaxanthin) have been shown to diminish oxidative stress and apoptosis, triggered by hyperglycemia in retinal tissues [176]. Future studies should determine the optimal dosage and routes of administration for these phytochemicals in the prevention or treatment of age-related eye diseases.

## 4. Conclusions and Implications for Future Research

In recent years, more attention has been paid to plant extracts used in the treatment of age-related eye diseases. Several polyphenols (such as anthocyanins, *Ginkgo biloba*, quercetin, and resveratrol) and carotenoids (such as lutein, zeaxanthin, and mezoxanthin) have shown significant preventive and therapeutic benefits against the aforementioned conditions. The involved mechanisms in these findings include mitigating the production of reactive oxygen species, inhibiting the tumor necrosis factor-*α* and vascular endothelial growth factor pathways, suppressing p53-dependent apoptosis, and suppressing the production of inflammatory markers, such as interleukin- (IL-) 8, IL-6, IL-1a, and endothelial leucocyte adhesion molecule-1.

Moreover, research into the mechanisms by which these compounds act and their role on molecular signaling indicates possible synergic interactions between compounds with complementary antioxidant and anti-inflammatory mechanisms of the two classes. The combination of bacterial carotenoids and some phenolic compounds has led to an increase in antioxidant activity of carotenoids [177]. On the other hand, the combination of lutein with certain polyphenols led to a decrease in absorption of lutein [178]. The combination of antioxidant compounds with complementary action mechanisms could be the key to optimizing the action of phytochemicals with antioxidant effect.

Research on synergistic combinations of carotenoids and polyphenols has shown that in certain combinations, polyphenols and carotenoids can interact synergically in the inhibition of several proinflammatory pathways. It has recently been discovered that these polyphenols of curcumin and carnosic acid produce (synergistically) the inhibitory effect of some carotenoids (lycopene, lutein, and beta-carotene) on the secretion of inflammatory mediators such as NO, TNF-alpha, and PGE2. In addition, synergism is significantly increased when polyphenol (curcumin or carnosic acid) combines with two of the aforementioned carotenoids; this synergistic effect occurs on the binary combinations of carnosic acid or curcumin and lycopene, beta-carotene, or lutein. At the same time, the synergistic anti-inflammatory effect that we are talking about is noticeable when carotenoids are present in combination with other polyphenols (quercetin, resveratrol, and gallic acid) [179, 180].

Consequently, future research should further establish and clarify the precise mechanism of action by which these compounds include extra and intracellular sites under healthy and pathological conditions. Knowing the mechanisms of action would enhance the acceptance and implementation of these compounds for use in clinical practice. Although beneficial effects of plant-based diets or phytochemicals on reducing the risk of chronic diseases have been shown through various epidemiological and in vitro/in vivo studies, much more mechanistic and clinical evidence is required to define a particular phytochemical as an inhibitor of specific cellular pathways or identify the plant-derived active compound with specific therapeutic properties. Moreover, researchers should further investigate the toxicological aspects of these phytochemicals and determine the optimal doses, routes of administration, and exact mechanism of action.

## Figures and Tables

**Table 1:** Effects of some polyphenols on age-related eye diseases in human studies.

Polyphenols	Natural source/dose	Cell type/type of study	Effects on chronic eye diseases	Ref	*p*
Anthocyanins	Bilberry	Retinal pigment epithelial cells (AREP 19)	Mediate a detergent-like perturbation of cell membranes and light-induced damage to the cell Inhibit AMD	[80]	

EGCG	10, 25, 50, 75, 100, and 150 *μ*M for 24 h	Human lens epithelial HLEB-3 cells	EGCG protects HLE cells from the mitochondria-mediated apoptosis (induced by H_2_O_2_ through the modulation of caspases, Bcl-2 family, and MAPK & Akt pathways) Cataract prevention	[92]	
	3 months	Human patients/placebo-controlled, double-blind, crossover design 34 patients: 18 ocular hypertension; 16 open-angle glaucoma	Neuroprotective function pattern-evoked electroretinograms increased in amplitude Favorable influence in inner retinal function in the eyes with early to moderately advanced glaucomatous damage	[94]	<0.01
	20 and 40 mM	Human RPE cell line ARPE-19	AMD and DR prevention Inhibitor of ocular angiogenesis and its vascular permeability Treatment and prevention of ocular angiogenic diseases: age-related macular degeneration, diabetic retinopathy, and corneal neovascularization	[95]	
	20 and 40 mM	Human retinal vascular endothelial cell (HREC) (case of diabetes)	Inhibits the expression of vascular endothelial growth factor (VEGF) It ameliorated the negative effect of high glucose concentrations on the cell viability and apoptotic rate The protective effects of EGCG under high-glucose conditions may be attributed to the regulation of inflammatory cytokines and inhibition of the MAPK/ERK-VEGF pathway	[96]	<0.01

*Ginkgo biloba*	40 mg GBE 2 times daily for 6 months	Human patients/experimental study, prospective, double blind	No significant changes were found in intraocular pressure and optic nerve head	[103]	>0.01
	Ginkgo biloba extract (40 mg, 3 times per day 4-week phases/8 weeks washout period)	Human patients/prospective, randomized, placebo-controlled crossover study	Changes in visual field and contrast sensitivity did not differ by treatment received or sequence	[104]	>0.2
	80 mg GBE 2 times daily	Human patients/retrospective study	Slowed the progression of visual field damage in patients with normal tension glaucoma	[105]	
	80 mg GBE orally, twice a day for four weeks	Human patients/prospective, randomized, placebo study	Desirable effect on ocular blood flow in normal tension glaucoma patients	[106]	
	40 mg, 3 times per day 4-week phases/8 weeks washout period/4 weeks of placebo treatment	Human patients/prospective, randomized, placebo-controlled, double-masked crossover trial	Improves preexisting visual field damage in some patients with normal tension glaucoma	[107]	
		Human patients/retrospective analysis	May be helpful in improving visual function in some individuals with NTG	[102]	
	Ginkgo biloba extract (EGB761) and ginkgolide B	Retina explants Three-dimensional tissue culture system	Protecting RGCs against apoptosis It decreased cellular apoptosis and inhibited caspase-3 activation, suggesting that ginkgolide B can promote RGC axon growth by protecting RGCs against apoptosis	[109]	

Quercetin	50 *μ*M	Cultured human RPE cells	AMD prevention It protects RPE cells from oxidative damage and cellular senescence in vitro in a dose-dependent manner Prevention of early AMD	[113]	<0.001
	50 *μ*M	Cultured human RPE cells (ARPE-19)	AMD prevention It protects human RPE cells from oxidative stress in vitro, via inhibition of proinflammatory molecules & direct inhibition of the intrinsic apoptosis pathway	[114]	

Resveratrol		Human lens epithelial cells	Cataract prevention It protects LECs from oxidative stress via the inhibition of the p53 pathway	[118]	
	5 and 10 mg/kg/day, 1 to 7 months	High-glucose culture Müller-treated cells	It is a therapeutic option to prevent diabetic retinopathy RSV (10, 20, and 30 mmol/L) significantly inhibited the HG-induced decreases in glutamate uptake, GS activity, GLAST, and GS expression	[124]	<0.05
	Longevinex	Human patients 3 clinical measures of visual function (Snellen visual acuity, contrast sensitivity, and glare recovery to a cone photostress stimulus)	Efficacy against AMD	[125]	

**Table 2:** Effects of some polyphenols on age-related eye diseases in animals.

Polyphenols	Natural source/dose/period	Cell type/animal model	Effects in chronic eye diseases	Ref	*p*	
Anthocyanins	Grape skin Asamurasaki-2 (AS2) (Japanese black rice) and chikushi-akamochi-2 (CH2) (Japanese red rice)	Sprague Dawley (SD) rat lens organ culture system	Inhibits selenite-induced cataractogenesis Inhibitory activities for lens opacity	[79]		
	Bilberry	Retinal pigment epithelium (RPE) cells	Inhibits AMD Exhibited antioxidant activity of variable efficiency at 9 anthocyanins tested Cells exhibited a resistance to the membrane permeabilization that occurs because of the detergent-like action of A2E	[80]		
	Blueberry; STZ, 60 mg/kg; and blueberry anthocyanins at 20, 40, and 80 mg/kg were given orally, 12 weeks	Rat retinas, male rats, 5 groups Streptozotocin-induced diabetic SD rats	It can protect retinal cells from diabetes-induced oxidative stress and inflammation, and this may be regulated through Nrf2/HO-1 signaling	[83]		
	Bilberry (*Vaccinium myrtillus*) extract 100 mg/kg, orally, 6 weeks	Streptozotocin-induced diabetic SD rats	Prevention of diabetic retinopathy using a dietary bilberry supplement	[84]		
	Seed coat of black soybean 50 mg/kg daily, orally, for 1, 2, and 4 weeks after intraperitoneal injection of N-methyl-N-nitrosourea (MNU)	Animal model of retinal degeneration N-methyl-N-nitrosourea-induced RD rats	Reduce retinal degeneration Anthocyanins (black soybean seeds) can protect retinal neurons from MNU-induced structural and functional damages, suggesting that it may be used as supplement to modulate RD	[85]		
	Maqui berry (*Aristotelia chilensis*)	Murine photoreceptor cells (661 W)	Suppress the light-induced photoreceptor cell death by inhibiting ROS production; the inhibition of phosphorylated-p38 may be involved in the underlying mechanism	[86]		
	Dried cornelian cherry (*Cornus mas* L.) polar, iridoid-polyphenol-rich fraction 1 kg of fresh fruit: 4.0 g anthocyanin fraction + 1.5 g of pure loganic acid Each animal was given 0.7% loganic acid or polyphenolic fraction, intraconjunctival administration of 1 drop (50 *μ*L)	New Zealand white rabbits, aged between 6-12 months, were used: 7 males and 7 females	Intraocular pressure (IOP)—hypotensive effect for loganic acid (0.7%)—could be compared with the widely ophthalmologically used timolol 25% decrease in IOP was observed within the first 3 hours of use	[87]		

EGCG	Green tea leaf extract (*Camellia sinensis*) In vivo, cataract was induced in 9-day-old rat pups by a single subcutaneous injection of sodium selenite The treated pups were injected GTL extract intraperitoneally prior to selenite challenge and continued for 2 consecutive days thereafter Cataract incidence was evaluated on the 16th postnatal day by slit lamp examination	Enucleated rat lenses	Inhibits selenite-induced cataractogenesis Green tea possesses significant anticataract potential and acts primarily by preserving the antioxidant defense system	[91]		
	Intraperitoneal (25 mg/kg) intraocular (5 *μ*L of 200 *μ*M)	Rat retinal neurons	Glaucoma prevention It provides protection to retinal neurons from oxidative stress and ischemia/reperfusion	[93]		
	20 and 40 mM	Animal models	Significantly reduced vascular leakage and permeability by blood-retinal barrier breakdown in VEGF-induced animal models Treatment and prevention of ocular angiogenic diseases: age-related macular degeneration, diabetic retinopathy, and corneal neovascularization	[95]		
	Nontoxic optimal concentration of EGCG used for the treatment of HCECs in vitro was 10 *μ*g/mL IL-1*β*, IL-6, IL-8, and TNF-*α* and was significantly inhibited in inflamed HCECs treated with 10 *μ*g/mL EGCG and 0.1% (*w*/*v*) HA (E10/HA) vs that in inflamed HCECs treated with either EGCG or HA alone	Rabbit DES model	Topical treatment with AT plus E10/HA increased tear secretion, reduced corneal epithelial damage, and maintained the epithelial layers and stromal structure The corneas of the E10/HA-treated rabbits showed fewer apoptotic cells, lower inflammation, and decreased IL-6, IL-8, and TNF-*α* levels In conclusion, we showed that AT plus E10/HA had anti-inflammatory and mucoadhesive properties when used as topical eye drops and were effective for treating DES in rabbits	[98]		

Ginkgo biloba	5 *μ*g of GBE, 4 times daily, for 14 days	Rabbits aged 7 weeks	Suppressed steroid-induced IOP elevation; it seems to prevent the adverse effects of DEX on TM cells	[100]		

Quercetin	Quercetin 10 *μ*M	Rat lens	Cataract prevention	[110]		
	The expression levels of BDNF, NGF, TrkB, synaptophysin, Akt, Bcl-2, cytochrome c, and caspase-3 using Western blotting techniques with and without QCT treatments were quantitated and compared with those of nondiabetic rats ELISA technique was used to determine the level of BDNF Caspase-3 activity and the level of glutathione were analyzed by biochemical methods	Diabetic rat retina	Significant increase in the level of neurotrophic factors and inhibited the level of cytochrome c and caspase-3 activity The level of an antiapoptotic protein Bcl-2 was augmented It may protect the neuronal damage in diabetic retina by ameliorating the levels of neurotrophic factors and by inhibiting the apoptosis of neurons Quercetin suitable therapeutic agent to prevent neurodegeneration in diabetic retinopathy	[112]		

Quercetin and resveratrol	0.01% QCT, 0.1% RES, 0.01% QCT + 0.1% RES (QCT + RES), or vehicle was topically applied	Desiccating stress (DS) mouse model	Reduced corneal staining in DS-exposed mice	[116]	QCT QCT + RES	<10^−3^ <0.05
			IL-1*α* tear concentration was reduced by QCT, RES, and QCT + RES compared to DS + vehicle mice		<0.05, 0.01, and 0.01	
			CD4^+^ T cells increased in recipients of DS-exposed mice and were lower in recipients of QCT- and RES-treated mice		<0.05, <0.05	
			The anti-inflammatory effect of QCT, RES, and QCT + RES on DED experimental model suggests that their topical application could be used for DED treatment			
	25 mg/kg/day quercetin by intraperitoneal injection daily, 2 months	Ccl2/Cx3cr1 double knockout (DKO) mice	Does not improve the retinal AMD-like lesions in the Ccl2^(−/−)^/Cx3cr1^(−/−)^ (due to its insufficient suppression of the inflammatory and apoptosis pathways in the eye)	[113]		
	Quercetin 33.63 mg/kg/day and chlorogenic acid	Pigmented rabbits	AMD prevention Its alleviating retinal degeneration	[115]	<0.05	

Resveratrol	40 mg/kg 3 groups: (1) normal saline—% 5 ethanol injected i.p. on postpartum day 10; (2) Na selenite—30 nmol/g body wt. injected s.c. on day 10; and (3) Na selenite—s.c. on day 10 + resveratrol (40 mg/kg) i.p. on days 10-13	48 SD rat lens	Inhibits selenite-induced cataractogenesis It suppressed selenite-induced oxidative stress and cataract formation in rats	[117]		
	25 *μ*M in DMSO, from Sigma, Saint Louis, MO	Porcine TM cells	Preventing or delaying of the abnormalities of the TM	[119]		
	4 months oral resveratrol administration (5 mg/kg/day)	Streptozotocin-induced diabetic Wistar rats	Therapeutic supplement to prevent from diabetic retinopathy It improves diabetic retinopathy possibly through the oxidative stress—nuclear factor kappa B—apoptosis pathway	[120]		
	1 month after the 5th injection of streptozotocin or buffer—20 mg/kg, daily, for 4 weeks, and all mice were killed 2 months after the injections	Streptozotocin-induced diabetic C57BL/6 mice	Prevent diabetic retinopathy It decreases vascular lesions and VEGF induction in mouse retinas of early diabetes	[121]		
	10 mg/kg 30 days	24 streptozotocin-induced diabetic Wistar albino male rats	Prevent diabetic retinopathy RSV suppressed the expression of eNOS, which is actively involved in the inflammation and healing process in chronic diabetes	[122]		
	5 mg/kg per day for 4 months	Streptozotocin-induced diabetic Wistar albino male rats	Prevent diabetic retinopathy Long-term resveratrol administration has beneficial anti-inflammatory properties in a rat model of diabetes	[123]		
	5 and 10 mg/kg/day, 1 to 7 months	Diabetic rat retina	Prevent diabetic retinopathy Significantly alleviated hyperglycemia and weight loss in diabetic rats Downregulated mRNA and protein expression of GLAST and GS in diabetic rat retina was prevented	[124]		

**Table 3:** Effects of some carotenoids on age-related eye diseases.

Carotenoid	Characteristics of the study	Cell type/animal model	Effects in chronic eye diseases	Ref.
Lutein and zeaxanthin	Multicenter, randomized, double-masked, placebo-controlled phase 3 study with a 2 × 2 factorial design (2006–2012) Participants were randomized to receive lutein 10 mg + zeaxanthin 2 mg, DHA 350 mg + EPA 650 mg, lutein + zeaxanthin and DHA + EPA, or placebo All participants were also asked to take the original AREDS formulation or accept a secondary randomization to 4 variations of the AREDS formulation, including elimination of beta-carotene, lowering of zinc dose, or both	4203 human patients, age 50-85 years	Median follow-up: 5 years, with 1940 study eyes, 1608 participants, progressing to advanced AMD Kaplan-Meier probabilities of progression to advanced AMD by 5 years were 31% (493 eyes, 406 participants), for placebo, 29% (468 eyes, 399 participants), for lutein + zeaxanthin, 31% (507 eyes, 416 participants), for DHA + EPA, and for lutein + zeaxanthin and DHA + EPA, 30% (472 eyes, 387 participants) Comparison with placebo demonstrated no statistically significant reduction in progression to advanced AMD (hazard ratio 0.90 (98.7% CI, 0.76-1.07); *p* = 0.12 for lutein + zeaxanthin; 0.97 (98.7% CI, 0.82-1.16); *p* = 0.70 for DHA + EPA; 0.89 (98.7% CI, 0.75-1.06); *p* = 0.10 for lutein + zeaxanthin and DHA + EPA) No apparent effect of beta-carotene elimination or lower-dose zinc on progression to advanced AMD More lung cancers were noted in the beta-carotene vs no beta-carotene groups (23 (2.0%) vs 11 (0.9%), nominal *p* = 0.04), mostly in former smokers Addition of lutein + zeaxanthin, DHA + EPA, or both to the AREDS formulation did not further reduce risk of progression to advanced AMD Because of potential increased incidence of lung cancer in former smokers, lutein + zeaxanthin could be an appropriate carotenoid substitute in the AREDS formulation	[152]
	Prospective study, in 1980 Repeated administration of a food frequency questionnaire during 12 years of follow-up	Human patients (female), age 45-71 years	Decrease the risk of cataracts, *p* = 0.04, foods rich in these carotenoids may decrease the risk of cataracts severe enough to require extraction	[155]
	Prospective study, in 1986 Repeated administration of carotenoids and other nutrients—frequency questionnaire during 8 years of follow-up	36644 human patients (male), age 45-75 years	May decrease the risk of cataracts severe enough to require extraction; this relation appears modest in magnitude As recommendations—to consume vegetables/fruit high in carotenoids daily; *p* = 0.03	[156]
	1354 people eligible, 246 developed a nuclear cataract (level 4 or 5 opacity) Nuclear opacity was assessed on a 5-point ordinal scale using lens photographs taken at baseline (1988-1990) and at follow-up (1993-1995) Food frequency questionnaire	Human patients, adults aged 43-84 years	Decrease the risk of nuclear cataracts Possible protective influence of lutein and vitamins E and C on the development of nuclear cataracts; the evidence provides weak support for these associations	[157]
	Usual nutrient intake = average intake from 5 food frequency questionnaires (were collected during a 13- to 15-year period before the evaluation of lens opacities). The duration of vitamin supplement use was determined from 7 questionnaires collected during this same period	478 human patients (nondiabetic female), aged 53-73 years	Prevention of age-related cataract The prevalence of nuclear opacities was significantly lower (*p* = 0.04) for women who used a vitamin C supplement for ≥10 years relative to women who never used vitamin C supplements (odds ratio, 0.36; 95% confidence interval, 0.18-0.72) Plasma measures of vitamins C and E taken at the eye examination were inversely associated with the prevalence of nuclear opacities	[158]
	Study for individual carotenoids and tocopherols in serum, quality-controlled HPLC method One-way ANOVA analysis and logistic regression analysis were applied	138 human patients with senile cataracts	No association The relation carotenoids-cataracts is biologically plausible; serum carotenoid levels are highly dependent on dietary intake and may not be clinically relevant biomarkers for cataract risk	[160]

Macular pigments	The optical density of MP was measured psychophysically	46 subjects, age 21-81 years with healthy maculae and in 9 healthy eyes known to be at high risk of AMD	Decrease the risk of AMD age-related decline (optical density of MP) at volunteers with no ocular disease (right eye: r(2) = 0.29, *p* = 0.0006; left eye: *r*(2) = 0.29, *p* < 0.0001) Healthy eyes (predisposed to AMD) had significantly less MP than healthy eyes at no such risk (Wilcoxon's signed rank test: *p* = 0.015) Supplemental lutein and zeaxanthin may delay, avert, or modify the course of this disease	[162]
	Lutein (L) and zeaxanthin (Z) extracted from each tissue sample were determined by HPLC	56 donors with AMD and 56 controls were cut into 3 concentric regions centered on the fovea	The results are inconsistent with a model that attributes a loss of L and Z in the retina to the destructive effects of AMD	[163]
	The relative risk for AMD—estimated according to dietary indicators of antioxidant status, controlling risk factors (smoking), by using multiple logistic regression analyses	356 case subjects, diagnosed with the advanced stage of AMD within 1 year prior to their enrollment, aged 55-80 years 520 control subjects	Increasing the consumption of foods rich in certain carotenoids (dark-green, leafy vegetables) may decrease the risk of developing advanced or exudative AMD	[165]

Lutein	Measuring macular pigment optical density (MPOD) and retinal sensitivity Vitamin supplementation (lutein 10 mg/day, zeaxanthin 2 mg/day), 3 months later—repeated ophthalmological examination	20 patients with medium/large drusen, 19 with RPD, and 15 control subjects	The mean MPOD significantly increased in RPD (*p* = 0.002), showing no more difference compared with controls (*p* = 0.3); no significant changes were found in mean retinal sensitivity and BCVA (*p* = 0.3 and *p* = 0.7) Medium/large drusen did not show significant changes on MPOD, retinal sensitivity, and BCVA (*p* = 0.5, *p* = 0.7, and *p* = 0.7, respectively) Different pathophysiology for RPD as compared with medium/large drusen	[168]

Lutein and zeaxanthin	Dietary intake	People over age 40 years (*n* = 8222)	No association with AMD Higher levels of lutein and zeaxanthin in the diet were related to lower odds for pigmentary abnormalities, one sign of early ARM (odds ratio among persons in high vs low quintiles = 0.1, 95% confidence interval: 0.1, 0.3) and of late ARM (odds ratio = 0.1, 95% confidence interval: 0.0, 0.9)	[170]
	Dietary intake + supplement intake of antioxidant vitamins and zinc at baseline and the 5-year incidence of early age-related maculopathy A 145-item food frequency questionnaire (FFQ) was used to assess nutrient intakes	During 1992-1994, 3654 people, aged > 49 years (82% of those are eligible) were examined for the Blue Mountains Eye Study baseline 5 years later, 2335 persons (75% of known survivors) were reexamined	No evidence of protection associated with dietary antioxidant or zinc intakes	[171]

Lutein + lycopene	Dietary intake Cross-sectional study Photodocumented retinal status and HPLC to measure plasma carotenoid concentrations	111 individuals with type 2 diabetes in a community-based study	Modulation of retinopathy risk A higher concentration of PVA carotenoids was associated with greater odds of diabetic retinopathy, after adjustment for risk factors (*p* = 0.049)	[172]

Zeaxanthin	Diet supplemented with 0.02% or 0.1% Zx	Age-matched normal rats	Inhibits diabetic retinopathy Zx supplementation has the potential to inhibit the development of retinopathy in diabetic patients	[173]

Lutein	Lutein, lutein + insulin 12 weeks	Streptozotocin-induced diabetic rats	Preventing the development of cataracts Treatment is useful in preventing the development of cataracts in streptozotocin-induced diabetic rats	[174]

Carotenoids and vitamins A, C, and E	Multicenter (5 ophthalmology centers in the USA) eye disease case control study	356 case subjects, diagnosed with an advanced stage of AMD within 1 year prior to their enrollment, aged 55 to 80 years, and residing near a participating clinical center The 520 control subjects were from the same geographic areas as case subjects, had other ocular diseases, and were frequency matched to cases according to age and sex	Dietary intake of carotenoids associated with a lower risk for AMD Adjusting for other risk factors for AMD—those in the highest quintile of carotenoid intake had a 43% lower risk for AMD compared with those in the lowest quintile (odds ratio, 0.57; 95% confidence interval, 0.35 to 0.92; *p* for trend = 0.02) Among the specific carotenoids, lutein and zeaxanthin, which are primarily obtained from dark-green, leafy vegetables, were most strongly associated with a reduced risk for AMD (*p* for trend = 0.001) Several food items rich in carotenoids were inversely associated with AMD A higher frequency of intake of spinach or collard greens was associated with a substantially lower risk for AMD (*p* for trend < 0.001) The intake of preformed vitamin A (retinol) was not appreciably related to AMD Neither vitamin E nor total vitamin C consumption was associated with a statistically significant reduced risk for AMD; a possibly lower risk for AMD was suggested among those with higher intake of vitamin C from foods Increasing the consumption of foods rich in certain carotenoids (dark-green, leafy vegetables) may decrease the risk of developing advanced or exudative AMD	[153]

## Abbreviations

AMD: Age-related macular degeneration
RBB: Retinal blood barrier
DME: Diabetic macular edema
DR: Diabetic retinopathy
EGCG: Epigallocatechin gallate
ERK: Extracellular signal-regulated kinase
GB: *Ginkgo biloba*
GSH: Glutathione
IL: Interleukin
IOP: Intraocular pressure
MP: Macular pigment
PDR: Proliferative diabetic retinopathy
ROS: Reactive oxygen species
RPE: Retinal pigment epithelium
VEGF: Vascular endothelial growth factor.
